# Effectiveness of Music-Based Intervention in Improving Uncomfortable Symptoms in ICU Patients: An Umbrella Review

**DOI:** 10.3390/ijerph182111500

**Published:** 2021-11-01

**Authors:** Yu-Fen Chen, Mei-Yu Chang, Lok-Hi Chow, Wei-Fen Ma

**Affiliations:** 1 Department of Public Health, China Medical University, Taichung 406040, Taiwan; yfchen1973@gmail.com; 2 Department of Nursing, Taichung Veterans General Hospital, Taichung 40705, Taiwan; cmy6040@vghtc.gov.tw; 3 Department of Anesthesiology, Taipei Veterans General Hospital, Taipei 112201, Taiwan; chowlh96@gmail.com; 4 School of Medicine, National Yang Ming Chiao Tung University, Taipei 112304, Taiwan; 5 Research Division, Center for Evidence-based Medicine, Taipei Veterans General Hospital, Taipei 112201, Taiwan; 6 Ph.D. Program for Health Science and Industry, China Medical University, Taichung 406404, Taiwan; 7 School of Nursing, China Medical University Hospital, Taichung 406040, Taiwan; 8 Department of Nursing, China Medical University Hospital, Taichung 404332, Taiwan

**Keywords:** intensive care unit, music-based intervention, uncomfortable symptoms, umbrella literature review

## Abstract

Background: Intensive care unit (ICU) patients experience multiple uncomfortable symptoms, which may be alleviated using music-based intervention, a nondrug treatment. This umbrella review aims to combine the data of systematic reviews and/or meta-analyses to evaluate the effectiveness of music-based intervention in improving uncomfortable symptoms in ICU patients. Methods: A comprehensive literature search was performed on the PubMed, Embase, Cochrane Library, Airiti Library, CINAHL, ProQuest, and Web of Science databases, and Epistemonikos. The search had no language restrictions, and articles on the improvement of symptoms using music-based intervention in adult ICU patients were included. This review protocol was registered on PROSPERO (CRD42021240327). Results: This umbrella review retrieved 5 systematic reviews and 41 original studies, including 39 randomized controlled trials, and 2 nonrandomized controlled trials. Diverse music was the most common music type used for music-based intervention, the intervention music was typically decided by the study participants (61%), and most subjects underwent one intervention session (78%). Furthermore, most music intervention sessions lasted for 30 min (44%). The positive results included decreased anxiety, decreased pain, decreased agitation, decreased anesthesia dose and sedative use, decreased chances of delirium, decreased feelings of uncomfort, and improved sleep quality. Conclusions: A systematic review on the effectiveness of music-based intervention in improving uncomfortable symptoms in ICU patients revealed that 20–30 min intervention sessions showed the best improvement in the uncomfortable symptoms in patients. This study provides a basis for using music-based intervention to relieve the uncomfortable symptoms in critically ill ICU patients, and a reference for empirical clinical practice.

## 1. Introduction

The intensive care unit (ICU) is a primary hospital unit for treating severe patients with life-threatening conditions [1]. ICU patients experience the fear of unpredictable death, multiple invasive tubes, and complex treatment procedures. Therefore, almost all patients experience one or more uncomfortable symptoms in the ICU [2]. Uncomfortable symptoms can be deconstructed as a combination of two concepts: “uncomfortable” and “symptoms”. “Uncomfortable” is defined as causing negative feelings (mental and physical) of unease and distress [3,4]. The word “uncomfort” is used to distinguish non-comfort from the in-the-state sense to the in-the-relief sense [5]. “Symptoms” are specific feelings experienced by the patient, owing to the disease, that causes the patient to further perceive deviations from the normal state [6,7]. Therefore, for this study, uncomfortable symptoms were defined as an individual’s subjective negative feelings, which encompass physiological and psychological symptoms.

Accordingly, the subjective feelings and behavioral presentation of an individual can be observed in the perception of changes from previously normal functions, and the frequency and severity of these symptoms can be used to determine the degree of distress [3,6]. These uncomfortable symptoms may persist until after discharge, resulting in a risk of short-term and long-term physical, cognitive, and mental suffering in discharged ICU patients [2,8]. Common uncomfortable symptoms observed in ICU patients include pain, anxiety, delirium, and sleep disorders. Multimodal therapy with drugs and nondrug treatments are vital in current clinical practice [2,9]. The systematic review (SR) of Thrane et al. [10] examined 32 randomized controlled trials on nondrug treatments in critically ill adult patients and found that the proportion of trials that employed music-based intervention to treat uncomfortable symptoms was 62.5% (23/32).

Music-based intervention is one of the most common nondrug treatments used by clinical staff and can effectively alleviate multiple uncomfortable symptoms [10]. The American Music Therapy Association defines music therapy as the use of personalized music listening as a treatment tool by health professionals that includes creating, singing, moving to, and/or listening to music to promote balance between physical, emotional, cognitive, and socialization needs to consequently improve communication barriers, release emotions, and promote physical recovery [11]. Furthermore, music-based intervention, a broader concept that incorporates both music therapy interventions and music medicine approaches [12,13], was used for this study. Music-based intervention stimulates the limbic system through pitch, rhythm, and melody, thereby stimulating the pituitary gland to release endorphins, leading to a sense of well-being [14]. This consequently affects physiological responses, such as changes in blood pressure (BP), body temperature, heart rate (HR), breathing, and muscle tension [10].

Music-based intervention is a nondrug treatment provided by health providers in the ICU setting and has a low risk of interfering with drugs and conventional physiological treatments that are currently used for treatment [10]. Many studies have demonstrated that music-based intervention can alleviate uncomfortable symptoms in patients [15]. However, with the exception of a retrospective umbrella analysis on pain [16], no studies have performed an appropriate integration and analysis of the effectiveness of music-based interventions in alleviating other uncomfortable symptoms in ICU patients. Hence, there is an absence of comprehensive understanding and adequate empirical evidence for the application of music-based intervention in relieving uncomfortable symptoms in ICU patients in clinical and research settings. Furthermore, the Society of Critical Care Medicine proposed pain, agitation/sedation, delirium, immobility (rehabilitation/mobilization), and sleep (disruption) (PADIS) guidelines in 2018 and recommended that music-based intervention be included in the nondrug multicomponent strategy to alleviate uncomfortable symptoms and relieve or decrease pain, anxiety, and sedative use, and promote sleep [2].

At present, most articles on the use of music-based intervention for uncomfortable symptoms in ICU patients cite noncombined study results or have a low level of evidence. Currently, there is no evidence summarizing the effectiveness of music-based intervention in decreasing uncomfortable symptoms in ICU patients, including the appropriate integration and analysis of music-based intervention types, and data on which uncomfortable symptoms observed in the ICU are suitable for treatment by music-based intervention. What is the evidence for supporting the effectiveness of music-based intervention in decreasing the uncomfortable symptoms in ICU patients? Therefore, this study aims to provide a comprehensive understanding of the uncomfortable symptoms that can be relieved using music-based intervention and further determine the requirements and recommendations for using music-based intervention for anxiety, pain, agitation, and other uncomfortable symptoms identified in empirical studies.

## 2. Methods

In this study, the umbrella review method [17] was used as the study design. Published SR articles on the effectiveness of music-based intervention in improving symptoms in adult ICU patients were compiled. Reporting was conducted according to the principles of the Preferred Reporting Items for Systematic reviews and Meta-Analysis (PRISMA) [18].

### 2.1. Protocol and Registration

The protocol for this umbrella review was originally registered with PROSPERO (http://www.crd.york.ac.uk/PROSPERO/, accessed on 6 April 2021) in April 2021 (Registration CRD42021240327).

### 2.2. Eligibility Criteria

This umbrella review included SR articles. There were four inclusion criteria: (1) SR articles focusing on adult patients in the ICU; (2) The intervention was a music-based intervention, including various types of music or natural sounds, either presented as a live performance or by listening; (3) The music-based intervention study design included a control group of routine care or non-music-based intervention as comparison; and (4) The effectiveness markers included improvement in any uncomfortable symptoms or physiological markers in the patient. There were two exclusion criteria: (1) Articles containing only a single study, and (2) Studies in which music-based intervention was performed only during surgery.

### 2.3. Search Strategy

Keywords and synonyms were established, P and I were used for the Boolean logic mixing of words, and no language restriction was used to search for SR articles published before 18 January 2021 on the PubMed, Embase, Cochrane Library, Airiti Library, CINAHL, ProQuest, and Web of Science databases, and Epistemonikos. The age range was limited to adults. The following presents the PubMed search strategy. The search strategy is presented in Table S1.

### 2.4. Study Selection

In this study, the EndNote (X9) software (Clarivate, Philadelphia, PA, USA) was used for data compilation, and the collected data was screened via the following steps. First, duplicate articles were excluded. Second, two of the authors (Y.-F.C. and W.F.-M.) independently screened the titles and abstracts of the included articles based on the study’s inclusion criteria. Moreover, the articles included and excluded by these two authors were consistent. Third, both authors (Y.-F.C. and W.-F.M.) independently assessed whether to include articles based on the entire paper; any disagreements were resolved by including a third author (L.-H.C.) in the discussion to reach a consensus.

### 2.5. Data Extraction

The piloted form template (as Table S2) was used for data compilation and included the aim, first author, year published, review design, number of studies included, participants, music types, outcomes reported, and the quality of each trial appraisal of each article. The data were extracted by one of the authors (Y.-F.C.), and discussion was conducted with the other two authors (L.-H.C. and W.-F.M.).

### 2.6. Quality Appraisal

The two authors who underwent empirical training (Y.-F.C. and W.-F.M.) assessed the quality of each review article using the Joanna Briggs Institute (JBI) Critical Appraisal Checklist for Systematic Reviews and Research Syntheses [19]; any disagreements were resolved by a third author (M.-Y.C.).

## 3. Results

### 3.1. Results of the Search Process

**Figure 1:**
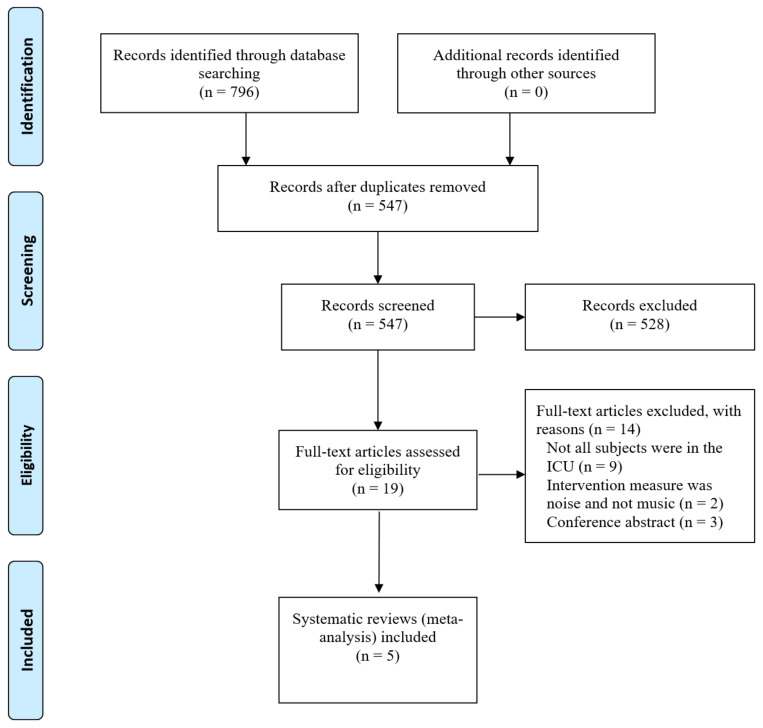
PRISMA flowchart (adapted from Moher et al., 2009 [18]).

A total of 796 articles were retrieved from the database search. After screening by title and abstract, we reviewed 19 full-text articles, of which 5 SR articles met the inclusion criteria [20,21,22,23,24]. The study selection PRISMA flow chart is depicted in Figure 1. A total of 41 original articles (as Table 1) were retrieved from the 5 SR articles [25,26,27,28,29,30,31,32,33,34,35,36,37,38,39,40,41,42,43,44,45,46,47,48,49,50,51,52,53,54,55,56,57,58,59,60,61,62,63,64,65]. A list of the excluded full-text studies with reasons is reported in Table S3. The PRISMA checklist result is reported in Table S4.

### 3.2. Description of Included SRs

The publication dates of the five SR articles were from 2014 to 2019; all were published in English. Two articles were meta-analyses [20,23], three focused on anxiety, accounting for most of the articles, two focused on pain, and one each examined other factors, such as the sedation and analgesia used, and the incidences of delirium, insomnia, and stress. The symptoms included in the two meta-analyses were anxiety [20] and pain [23]. Two meta-analyses showed that one 20–30-min music intervention session could alleviate pain in ICU patients. The five SR articles collected and integrated 6–18 articles each. The original articles used these tools for critical appraisal: two presenting quality using Grading of Recommendations Assessment, Development and Evaluation (GRADE) [20,21], one assessing risk of bias (ROB) [23], one employing the Physiotherapy Evidence Database (PEDro) scale for evaluation [24], and one presenting the American Association of Colleges of Nursing’s evidence leveling hierarchy [22]. Further details are presented in Table 2.

The publication date range of the 41 original articles was from 1995 to 2018. Of these, 39 were randomized controlled trials and 2 were nonrandomized controlled trials [31,43]. The original studies were from 11 countries (Australia, Canada, China, France, Germany, Iran, Netherlands, Spain, Taiwan, Turkey, and USA), had 10–373 enrolled subjects, and included adults in internal medicine and surgical ICUs, with/without ventilator use, who were critically ill after surgery. The physiological parameters and psychological status results were obtained via data analysis. The physiological markers included noninvasive measurements, such as BP, diastolic blood pressure (DBP), HR, respiratory rate (RR), and systolic blood pressure (SBP), and invasive measurements, such as blood stress biomarkers. Positive symptom results included decreased anxiety, decreased pain, decreased agitation, decreased anesthesia dose and sedative used, decreased chance of delirium, decreased feelings of uncomfort, and improved sleep quality.

Of the 41 studies on ICU patients, the study subjects had the levels of consciousness of awake, alert, or self-report in 23 studies (56.1%), Ramsay scores (2–4 points) were employed in 1 study, Glasgow Coma Scale scores ≥9 were employed in one study, and the level of consciousness was not indicated in other studies. With regard to the improvements in symptoms after music intervention measures, 19 (46.3%) studies reported statistically significant improvements, and 9 (21.9%) studies reported no statistically significant difference. However, 13 (31.7%) studies did not mention symptom improvement results. Although two (4.9%) studies contained physiological parameter data, the physiological parameters were limited to BP, HR, RR, and oxygen saturation (SpO_2_), and did not clearly state whether there was a direct correlation between the physiological parameters and the anxiety symptoms [31,47]. From the aforementioned results, it can be observed that there were no statistically significant differences when the number of sessions (≥2) and (1) were used for stratification to compare symptom improvement effectiveness (present/absent) (X2 = 0.006, *p* = 0.657).

### 3.3. Summary of Music-Based Interventions Characteristics

Of the 41 articles retrieved from the literature review, most employed music-based intervention as the main objective of relaxation and used diverse music as the music type (Table 3). In these studies, the subjects that decided the intervention music were the study participants in 25 (61%) studies, researchers in 10 (24%) studies, music therapists or music players in 4 (10%) studies and were not described in 2 (5%) studies. Most studies (n = 32, 78%) had one intervention session; three had two intervention sessions (7%); and six had ≥3 intervention sessions (14.6%). Most studies (n = 18, 44%) had an intervention session of 30 min; 15 (37%) had an intervention session of >40 min; 5 (12%) had an intervention session of 20–25 min; and 3 (7%) had an intervention session of ≤15 min. With the exception of three studies in which there were live performances, all experimental groups wore headphones (n = 31, 76%), or earphones (n = 5, 12%), one study used music pillows, and one study did not provide information on this aspect. The subjects in the control group wore headphones (n = 16, 39%), or earphones (n = 1, 2.4%), or rested (n = 6, 14.6%), and one study did not provide information on this aspect. Other control groups received routine care (n = 17, 41.5%) in which headphones or earphones were not worn.

### 3.4. Critical Appraisal of Included SRs

Table 4 summarizes the JBI questionnaire for the critical appraisal of SRs that was used to evaluate the quality of the studies [19]. All included review studies obtained >7 “Yes” (positive) results on the quality checklist. Studies by Bradt and Dileo [20] and Richard-Lalonde et al. [23] obtained “Yes” for all 11 items. However, some studies were assessed as “No” or “Unclear” because of publication bias, reduced data extraction, and quality appraisal errors.

## 4. Discussion

This umbrella review summarized five SRs and meta-analyses and included data from 41 original studies on the effectiveness of music-based intervention in improving symptoms in ICU patients. Overall, the evidence supporting the effectiveness of music-based interventions in improving symptoms was weak. The symptoms examined in the five SRs were anxiety, stress, pain, insomnia, sedation and analgesia, and reduction in delirium incidence, but only two meta-analyses showed that music-based intervention could improve anxiety [20] and pain [23] and concluded that music-based intervention was beneficial in reducing the anxiety of mechanically ventilated patients, and that one 20–30-min music intervention session could alleviate pain in ICU patients. Considering the quality of these studies, two meta-analyses were considered high-quality papers based on the JBI scale, and the other three articles were considered to be of moderate quality. The JBI scale is extremely simple and easy to use and can aid in determining the content that should be included in systematic studies.

### 4.1. Music-Based Interventions

Overall, music-based intervention is a generally accepted method and is used in different regions, cultures, and study categories (Table 3). Relaxing music was the most common choice of music and there were some live performances as well. However, in scientific studies and clinical settings, important intervention measures can be used repeatedly. Therefore, it is important for the intervention measures to be clear and specific, such that the principles are reproducible. The principles for music intervention are summarized as follows: (1) Relaxing music (classical, nature-based sounds, jazz, and country music) [20,21,24]; (2) Music with similar background considerations and cultures according to regions (Chinese, religious, and reed flute) [20,23,24]; (3) Slow tempo of 60–80 beats per min (bpm), with the primary purpose of decreasing the HR [20,23]; (4) Mainly one intervention session [20,21,22,23,24]; (5) The patient is able to make choices regarding Principles (1) and (2), achieving autonomy and respect, and enabling the subject to have his/her own views on music-based interventions [21,23]. Other studies emphasized the importance of this theme [49,66,67].

There were large differences in the intervention durations presented in the data, ranging from 10 to 240 min. Only the meta-analysis of Richard-Lalonde et al. [23] found that 20–30 min of music intervention was effective, but this study only analyzed pain. Hence, it can be seen that there is great heterogeneity in music-based interventions, and without integrated results with empirical significance, it is difficult to provide evidence that can be used as a clinical index. We recommend that future SRs combine durations, wherein subgroups can be used to present durations if the range and heterogeneity are large. Concomitant anesthesia or sedative use is a confounding factor for the effectiveness of music-based interventions. Although anesthesia or sedative use is unavoidable in the ICU, almost all the included studies were randomized controlled trials, and the only solution was to control for this confounding factor during analysis. The use of headphones, earphones, and music pillows suggests the possibility of placebo. However, control groups were often referred to as standard care (SC) in most SRs. We recommend that placebo be used in future studies on music-based interventions, as SC suggests that the effect of music-based interventions is nonpurified and uncontrolled.

### 4.2. Symptoms

When examining symptom alleviation in ICU patients by music-based intervention, diverse symptoms were examined (Table 2), including anxiety [20,22,24], pain [22,23], insomnia [22], sedation, analgesia, delirium [21], and stress [24]. With regard to the SRs with the same theme, the earliest paper was published by Bradt and Dileo [18] in the Cochrane Library in 2014. This study examined the effectiveness of music-based interventions in mechanically ventilated ICU patients. Following that, four similar articles were successively published between 2017 and 2020. However, the meta-analysis results were only presented for anxiety and pain, and the effectiveness of music-based interventions for insomnia, sedation and analgesia use, and the incidence of delirium was presented by the results of single studies. Further studies are warranted to obtain a comprehensive knowledge of the improvements in the uncomfortable symptoms of ICU patients by music-based interventions.

Symptoms, such as anxiety and pain, are subjective experiences, and the frequency, severity, and degree of distress of the symptoms perceived by individuals require subjective experiences for evaluation. However, out of the 41 studies compiled from the SRs included in this study, only 23 studies (56.1%) presented the consciousness status of study subjects (awake, alert, or had self-report ability), indicating that these study subjects possessed the ability to express subjective feelings during the study evaluation. Therefore, the choice of evaluation tool is an important consideration when the evaluation tool and the consciousness state of the subject are related. Psychological symptoms are important markers of care quality in patients, and studies need to overcome the blind spots of the measurement tools.

With regard to anxiety, three SRs examined the effectiveness of music-based interventions on anxiety [20,22,24], and most showed evidence that music-based interventions were effective in treating anxiety. However, only Bradt and Dileo [20] performed a meta-analysis on anxiety, and they reported that music-based interventions decreased the mean state anxiety by 1.11 standard deviation units, compared with routine treatment (95% CI, −1.75 to −0.47, *p* = 0.0006). In addition, two SRs, published in 2014, only listed or described the positive or negative effects on anxiety, without performing a subgroup analysis. The tools used for the subjective assessment of anxiety severity were the Spielberger State-Trait Anxiety Inventory, the Visual Analog Scale (VAS), and the Faces Anxiety Scale. With regard to the physiological parameters, HR, respiration, BP, SpO_2_, and serum and urine cortisol concentrations (Table 1) were mainly monitored.

With regard to pain, two SRs examined the effectiveness of music-based interventions on pain [22,23]. Meghani et al. [22] included two studies, of which one showed that music-based interventions could effectively alleviate pain, whereas the other reported no significant difference. Richard-Lalonde et al. [23] employed subgroup analysis to address high heterogeneity and their analyses revealed that 20–30 min of music-based interventions had the greatest effectiveness in decreasing pain. Overall, the evaluation of pain severity was mainly based on scales, such as the Numeric Rating Scale, the VAS, the Behavioral Pain Scale, the Critical-Care Pain Observation Tool, the Thermometer Visual Pain Scale, The University of California at Los Angeles Universal Pain Assessment Tool, and the Verbal Pain Intensity Scale. These scales are short and easy to use for evaluation. However, the physiological monitoring of pain was rarely present in the SRs that evaluated pain. In contrast, the study by Korhan [47] presented physiological parameters but did not provide the evidence of physiological parameters related to psychological responses. For example, decreased HR cannot be the only marker of decreased anxiety. Therefore, we recommend that studies measure both the physiological and psychological aspects when using symptoms as study variables in order to obtain valid and reasonable deductions.

### 4.3. Agitation and Sedation

Agitation is often observed in critically ill patients in the ICU. However, many agitation-related studies are not included when assessing the concept of uncomfort [25,56,64,68]. Therefore, we recommend that a search and analysis on the SRs reporting that music-based interventions can improve agitation in ICU patients be performed. In addition, sedation is often observed in studies on music-based interventions for critically ill patients in the ICU [64]. However, this study found that sedation and agitation in patients are highly correlated, and that both are two related ends of a scale. Examples include the Richmond Agitation Sedation Scale (RASS) [69] and the Ramsay Sedation Scale [70]. Therefore, although sedation is not an uncomfortable symptom, we recommend that it be simultaneously considered and analyzed when examining agitation in the SRs on music-based interventions for critically ill patients in the ICU.

This study found that, although insomnia [22] and delirium [21] were included in five SRs, a comprehensive study conclusion is still lacking. However, the evidence of a direct connection between insomnia and delirium and uncomfortable symptoms is relatively weak, and the severity of insomnia and delirium often shows a positive correlation with fatigue [71,72]. Although fatigue satisfied the criterion of an uncomfortable symptom, it was not searched as an uncomfortable symptom in this study on music-based interventions in critically ill patients in the ICU and was only noted in a meta-analysis on fatigue in cancer patients [73]. Therefore, we recommend that studies on fatigue-related uncomfort should be included in future studies on music-based interventions in critically ill patients in the ICU.

### 4.4. Methodology

In this study, we found that there were many study method restrictions in the five SRs. For example, most of the data contained text descriptions, and tables with clear categories were not provided, leading to incomplete results. Furthermore, the study design of a high proportion of the control groups only stated the SC and did not clarify the actual intervention device. As there were differences in the comfort and functions of noise-canceling earphones, headphones, or earphones [74,75], the study results tended to be affected by the differences in intervention devices. However, a clear description of the intervention measures was lacking. In addition, there were limitations in blinding participants and assessors in music-based interventions. Although Yaman Aktaş et al. [64] used music pillows to increase the feasibility of blinding the caregivers and/or assessors, it was difficult to effectively conduct a blinded music-based interventions trial on participants, caregivers, and/or assessors. Moreover, all the five SRs assessed the RCT quality using different evaluation tools. Only three articles provided GRADE recommendation grades [20,21,23]. Although the evaluation tools used were different, recommendation grades should be provided for readers to be able to make consistent quality judgments.

This umbrella review comprehensively compiled the results of music-based interventions intervention measures in alleviating the uncomfortable symptoms of ICU patients that were reported in existing SRs. It was found that the SRs included in this study mostly included RCTs. Although the strength of RCTs is that they can present causality for the effectiveness of music-based interventions, only two SRs presented meta-analysis results. After searching, we found that the first meta-analysis on the effects of music-based intervention measures on anxiety was published in 2014 [20]. Thus, it can be seen that the study method for the use of music-based interventions measures on anxiety is almost perfect. However, the SRs published after 2014 did not provide meta-analysis data [23,24], which is why more concrete empirical data on the effectiveness of music-based interventions in decreasing anxiety cannot be provided, even though more study data was generated. Even though the results of the SRs included in this study employed diverse scales for symptom assessment, which led to difficulty in simultaneously including these scales in the meta-analysis, Richard-Lalonde et al. [23] obtained a good cutoff point for evaluating the effectiveness of music-based interventions in alleviating pain in ICU patients. Therefore, we recommend that the SR classification be specific, rational, concise, and comprehensive, and provide meta-analysis results as much as possible so that true and complete meta-analysis data can be used for subsequent umbrella studies, or as guidelines for a quick understanding of the information on the effectiveness of music-based interventions in clinical applications.

### 4.5. Limitations

There were limitations in the evaluation of the physiological parameters because appropriate judgments could not be made owing to incomplete data (direct evidence and correlation between physiological parameters and anxiety, stress, or pain was weak). For example, changes in the HRs of ICU patients may be caused by sedatives, inflammation, or sepsis [76], and an increased HR may not necessarily represent increased anxiety or stress. Therefore, we were unable to use the results of the physiological parameter evaluation for deductions in this study. Furthermore, although this study did not use language and time as exclusion criteria when searching for SRs, the search results mostly comprised articles published in English. In addition, the search spectrum in this study was limited to seven databases and we were unable to conduct a database search for other regions. Therefore, we were unable to determine if SRs published in languages other than English were overlooked in this study, or if only SRs in English were published worldwide. This may have limited the interpretations established in this study.

## 5. Conclusions

This umbrella review included and analyzed five SRs on the effectiveness of music-based interventions in improving uncomfortable symptoms in ICU patients. It was found that diverse music was the most common music type used, and that the main objective was to achieve relaxation in order to alleviate uncomfortable symptoms in ICU patients. Although a meta-analysis of the intervention methods was lacking, a single intervention session of 20–30 min showed the best improvement in the uncomfortable symptoms in patients. This study provides a clear basis for using music-based interventions to relieve uncomfortable symptoms in critically ill ICU patients, and a reference for empirical clinical practice.

## Figures and Tables

**Table 1:** Included reviews and randomized controlled trials.

Author, Year	Physiological Parameters					Symptoms		
	SBP	DBP	HR	RR	SpO_2_	Pain	Anxiety	Other
Aghaie, 2014 [25]							FAS (+)	RASS (+)
Ames, 2017 [26]						NRS (+)		
Beaulieu-B, 2013 [27]	−		−	−				Serum cortisol (+)
Blankfield, 1995 [28]								Depression (−)
Broscious, 1999 [29]						NRS (−)		
Chan, 2007 [30]			+	+	+	UCLA (+)		
Chan, 2009 [31]	+	+	+	+				
Chiasson, 2013 [32]						TVPS (−)		
Chlan, 1995 [33]	−	+	+	+	−			Mood states (+)
Chlan, 1998 [34]			−	−			STAI (+)	
Chlan, 2007 [35]			+					Biomarkers ^§^ (−)
Chlan, 2013 [36]							VAS (+)	Urine cortisol (+)
Ciğerci, 2016 [37]						VAS (+)	STAI (−)	
Conrad, 2007 [38]			−					Sedation level (+)
Cooke, 2010 [39]						NRS (−)	FAS (−)	Discomfort: NRS (−)
Dijkstra, 2010 [40]	−	−	−	−				Sedation level (+)
Guilbaut, 2017 [41]						NRS (+)		
Han, 2010 [42]	+	+	+	+	−		STAI (+)	
Hunter, 2010 [43]			+	+			98% less ^†^	
Iblher, 2011 [44]	−	−	−		−			Delirium: CAM (−)
Jaber, 2007 [45]	−	−	−	−		NRS (+)		
Jafari, 2012 [46]						NRS (+)		
Korhan, 2011 [47]	+	+	−	+	−			
Kyavar, 2016 [48]						CPOT (+)		
Lee, 2017 [49]	+	−	+				STAI/VAS (+)	Serum cortisol (+)
Lee, 2005 [50]	−	−	+	+			STAI (−)	
Mateu-C, 2019 [51]						BPS (−)		
Sanjuan N, 2013 [52]						NRS (−)	STAI (+)	
Özer, 2013 [53]					+	VPIS (+)		
Phillips, 2007 [54]			−	−	−			
Saadatmand, 2015 [55]						VAS (+)		
Saadatmand, 2013 [56]	+	+					FAS (+)	RASS (+)
Shultis, 2012 [57]						VAS (−)		
Su, 2013 [58]			+	+				Sleep quality (+) ^‡^
To, 2013 [59]	−		−	−				Ramsay scores (−)
Voss, 2004 [60]						VAS (+)		
Wong, 2001 [61]				−			STAI (+)	
Wu, 2008 [62]	−	−	−	+	−		VAS (+)	
Yaghoubinia, 2016 [63]						BPS (+)		
Yaman A, 2016 [64]						CPOT (+)		
Yarahmadi, 2018 [65]						VAS (−)		

BPS = Behavioral Pain Scale; CAM = Confusion Assessment Method for the ICU; CPOT = Critical-Care Pain Observation Tool; DBP = diastolic blood pressure; FAS = Faces Anxiety Scale; HR = heart rate; NRS = Numeric Rating Scale; RASS = Richmond Agitation Sedation Scale; RR = respiratory rate; SBP = systolic blood pressure; SpO_2_ = oxygen saturation; STAI = Spielberger’s State-Trait Anxiety Inventory; TVPS = Thermometer Visual Pain Scale; UCLA = The University of California at Los Angeles Universal Pain Assessment Tool; VAS = Visual Analog Scale; VPIS = Verbal Pain Intensity Scale; † = Patient self-assessment of anxiety (six items ranked on a Likert scale); ‡ = Sleep: VSHS(+), N2(+), N3 (+); § = Levels of four biomarkers of stress response: Epinephrine, Norepinephrine, Corticotropin, Cortisol; Shaded cells = Not clearly stated as SR, but separately confirmed by the authors of this paper; “+” = symptoms positive statistically significant improvement; “−” = no significant difference.

**Table 2:** Summary of included reviews.

First Author/ Year/ Review Design	Objective	Included Studies (Range)	Number of Participants (Range)	Countries	Study Designs	Settings	Symptoms/ Phenomena of Interest	Positive Outcomes Related to Symptoms	Summary of Critical Appraisal
Bradt and Dileo, 2014 [20] Systematic review and meta-analysis	Effects of music therapy on anxiety and other outcomes in mechanically ventilated patients	14 (1995–2013)	912 (10–373)	5 USA 3 China 1 Canada 1 Germany 1 France 1 Netherlands 1 Taiwan 1 Turkey	all RCTs	MV patients in ICU, LCAT, or “step-down” unit All participants were alert All adults	- anxiety - physiological parameters	^ reduction anxiety ^ relaxation response (↓ RR and SBP)	Quality of the evidence (GRADE): low
Gonzalo Garcia et al., 2019 [21] Systematic review	Efficacy of music in providing sedation and analgesia and reducing the incidence of delirium in critically ill patients	6 (1995–2017)	734 (41–373)	3 USA 2 Canada 1 Turkey	all RCTs	With or without MV patients in ICU All adults	- sedation and analgesia used - incidence of delirium	1 ↓ sedation requirements	Quality of the evidence (GRADE): low
Meghani et al., 2017 [22] Integrative review	Effects of music on symptom management of anxiety, pain, and insomnia in critically ill patients	9 (2010–2017)	943 (17–373)	2 Iran 2 Turkey 2 USA 1 Australia 1 China 1 Taiwan	7 RCTs 1 Quasi 1 Feasibility study with historical controls	Critically ill patients in critical care settings 7 MV support 1 Open-heart 1 Medical ICU All adults	- pain - insomnia - anxiety	1 ↑ sleep quality 3 ↓ anxiety 1 ↓ agitation levels 1 ↓ discomfort 2 ↓ HR and RR 1 ↓ SBP and DBP 1 ↓ RR, SBP, and DBP 1 ↓ HR, RR, BP, and anxiety	American Association of Colleges of Nursing’s evidence leveling hierarchy 6 of level B ^†^ 3 of level C ^‡^
Richard-Lalonde et al., 2020 [23] Systematic review and meta-analysis	Effects of music interventions on pain in adult ICU patients	18 (1999–2018)	1173 (17–156)	5 Iran 5 USA 2 France 2 Spain 2 Turkey 1 Australia 1 China	all RCTs	Patients in ICU All adults	- pain	^ 20–30 min of music reducing pain	Risk of bias 2 high risk of random sequence 2 high risk of allocation concealment 18 high risk of performance 16 high risk of detection bias 2 high risk of attrition bias
Umbrello et al., 2019 [24] Systematic review	Effects of music therapy in reducing stress and anxiety in critically ill patients	11 (1998–2017)	959 (17–373)	4 China 2 USA 2 Taiwan 1 Australia 1 Netherlands 1 Turkey	10 RCTs 1 Quasi	Patients in ICU All adults	- stress - anxiety	6 ↓ anxiety 2 ↓ HR and RR 2 ↓ HR, RR, SBP, and DBP 1 ↓ HR 1 ↓ RR and BP 1 ↓ HR, RR, and BP 1 ↑ level of sedation 1 ↓ RR, SBP, and DBP 1 ↑ sleep quality 1 ↓ sedative exposure	Assessment by using PEDro Scale 7: 8 in 11 2: 7 in 11 2: 6 in 11

BP = blood pressure; DBP = diastolic blood pressure; GRADE = Grading of Recommendations Assessment, Development and Evaluation; HR = heart rate; ICU = intensive care unit; LCAT = long-term acute care at hospital; MV = mechanical ventilation; PEDro = Physiotherapy Evidence Database; RCT = randomized controlled trial; ROB = risk of bias; RR = respiratory rate; SBP = systolic blood pressure; ^ = result of pooling data from meta-analysis; † = well-designed controlled studies; ‡ = randomized controlled trials with inconsistent results.

**Table 3:** Music-based intervention characteristics.

Author, Year	Music Type	Duration (Min)	Timing/ Setting	Session/Day	Music Selection	Delivery	Comparator	Conscious/Self-Report Ability	Symptoms
Aghaie, 2014 [25]	N-BS	20	Weaning MV	1	R	HP	NRH		Anxiety (+) Agitation level (+)
Ames, 2017 [26]	MusiCure Dreams Album	50	Any time	4–8	R	HP	SC	Yes	Pain (+) Opioid use (−)
Beaulieu-Boire, 2013 [27]	Classical	60	Day	2	MT	HP	NRH		Sedation intensity (−)
Blankfield, 1995 [28]	New Age Relaxing	30	Day	2	R	HP	SC		Opioid requirement (−)
Broscious, 1999 [29]	Ten Categories of Cassettes	10	Procedure: CTR	1	P	Earphones	WNH, SC	Yes	Pain (−)
Chan, 2007 [30]	Three types	45	Procedure: C-Clamp	1	P	Earphones	SC	Yes	
Chan, 2009 [31]	Classical, Religious, and Jazz	30		1		HP	No control group	Alert	
Chiasson, 2013 [32]	Harpist’s Choice	10	Rest	1	Music Player	Live Harp	SC	Yes	Pain (−)
Chlan, 1995 [33]	Helen Bonny	30		1	P	HP	NRH		
Chlan, 1998 [34]	Classical, New Age, Country, Religious	30		1	P	HP	Quiet Rest	Alert	Anxiety (+)
Chlan, 2007 [35]	Classical, New Age, Country	60		1	P	HP	Quiet Rest		
Chlan, 2013 [36]	Self-initiated Preferred Music	79.8 (mean)	Day and Night	Diversity	P	HP	NCH, SC	Alert	Anxiety (+) Sedative exposure (+)
Ciğerci, 2016 [37]	Folk or Classical	30	Rest	2	P	HP	SC	Yes	
Conrad, 2007 [38]	Mozart Piano Sonatas	60		1	R	HP	NRH		
Cooke, 2010 [39]	Classical, Jazz, Country, Western, New Age, Easy Listening, “Other”	15	Procedure: Turning	1	P	Earphones	NRE	Yes	Pain (−), Anxiety (−), Discomfort (−)
Dijkstra, 2010 [40]	Classical and Easy Listening	30		3 (2 days)	P	HP	Rest	Ramsay Score: 2–4	Higher level of sedation (+)
Guilbaut, 2017 [41]	Music Care Selection	20	Dressing, ETS, Turning	1	P	HP	NRH	Yes	Pain (+)
Han, 2010 [42]	Relaxation	30		1	P	HP	NRH, Rest	Alert	Anxiety (+)
Hunter, 2010 [43]	Patient-Tailored Live Music	45–60	Weaning Trials	3 times/week	Patient-tailored	Live Music	SC (historical controls)		
Iblher, 2011 [44]	Classical, Baroque	60	Day	1	R	HP	SC		CAM (−)
Jaber, 2007 [45]	U-Shaped Montage	20	Rest	1	P	HP	SC	Yes	
Jafari, 2012 [46]	A List Provided By A Music Expert	30	Rest	1	P	HP	NRH	Yes	Pain (+)
Korhan, 2011 [47]	Classical	60		1	R	HP	SC	GCS ≥ 9	
Kyavar, 2016 [48]		30	Dressing Change	1	P	HP	NRH		
Lee, 2017 [49]	Classical, Natural Sounds	30	4–4.30 pm	1	P	HP	NRH	Yes	Anxiety (+)
Lee, 2005 [50]	Classical, Religious, Natural Sounds	30		1	P	HP	NRH	Alert	Anxiety (−)
Mateu-Capell, 2019 [51]	Reikid Merlin’s Magic	60	Rest	1	MT	HP	NCH		
SanjuanNaváis, 2013 [52]	Researchers’ Selection	30	Rest	3–5 Minimum/8 h	P	Earphones	SC	Yes	
Özer, 2013 [53]	Patients’ Selection	30	POD 1	1	P	Earphones	Rest		Pain (+)
Phillips, 2007 [54]	Live Music	25		1	P	Live Music	Quiet Rest		
Saadatmand, 2015 [55]	CDs	30–90	Rest	1	P	HP	NRH	Yes	Pain (+)
Saadatmand, 2013 [56]	N-BS	30–90		1		HP	NRH		Anxiety (+) Agitation level (+)
Shultis, 2012 [57]	Researcher-Compiled CDs	22 (mean)	Rest	1	P	CD Player	SC	Yes	Pain (−)
Su, 2013 [58]	Noncommercial Music	45	Nocturnal Sleep Time	1	R	HP	SC	Clear	Sleep quality (+)
To, 2013 [59]	Classical	240	Day	1	R	HP	NRH		Success of sedation vacation (−)
Voss, 2004 [60]	Six Types	30	Procedure: Chair Rest	1	P	HP	SC	Yes	Pain (+)
Wong, 2001 [61]	Various Chinese and Western music	30		1	P	HP	Rest	Alert	Anxiety (+)
Wu, 2008 [62]	Classical, Orchestral, Religious, New Age, Hymn	30		1	P	HP	SC		Anxiety (+)
Yaghoubinia, 2016 [63]	Beach Walk by Arnd Stein	30	Rest	1 (total 3 days)	R	HP	SC		
YamanAktaş, 2016 [64]	Reed Flute	20 pre- and 20 post-ETS	Procedure: ETS	1	R	Music Pillow	SC	Yes	
Yarahmadi, 2018 [65]	15 Pieces	15 pre- and 15 post-CTR	Procedure: CTR	1	P	HP	SC	Yes	Pain (−)

CAM = confusion assessment method; CD = compact disc; CTR = chest tube removal; ETS = endotracheal suction; GCS = Glasgow Coma Scale; HP = headphones; MT = music therapist; MV = mechanical ventilation; N-BS = nature-based sounds; NCH = noise-canceling headphones; NRE = noise reduction via earphones; NRH = noise reduction via headphones; P = participant; POD = postoperative day; R = researcher; SC = standard care; WNH = white noise headphones.

**Table 4:** Critical appraisal of included systematic reviews.

Citation/Questions	Q1	Q2	Q3	Q4	Q5	Q6	Q7	Q8	Q9	Q10	Q11
Bradt and Dileo, 2014 [20]	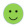	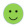	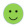	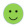	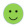	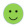	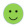	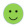	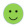	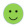	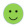
Gonzalo Garcia et al., 2019 [21]	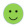	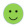	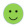	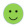	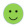	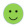	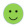	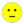	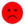	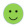	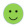
Richard-Lalonde et al., 2020 [22]	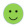	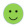	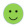	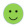	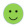	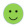	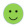	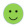	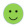	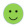	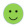
Umbrello et al., 2019 [23]	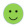	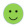	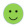	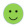	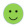	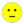	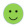	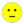	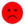	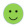	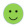
Meghani et al., 2017 [24]	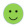	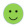	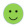	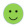	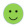	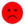	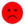	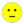	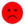	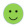	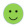

N = No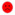; NA = Not applicable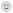; U = Unclear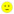; Y = Yes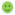. Source: Joanna Briggs Institute (2020) [19]. Items by each criterion (question number): Q1: Was the review question clearly and explicitly stated?; Q2: Were the inclusion criteria appropriate for the review question?; Q3: Was the search strategy appropriate?; Q4: Were the sources and resources used to search for studies adequate?; Q5: Were the criteria for appraising studies appropriate?; Q6: Was the critical appraisal independently conducted by two or more reviewers?; Q7: Were there methods to minimize errors in data extraction?; Q8: Were the methods used to combine studies appropriate?; Q9: Was the likelihood of publication bias assessed?; Q10: Were recommendations for policy and/or practice supported by the reported data?; Q11: Were the specific directives for new research appropriate?

## Data Availability

This study did not report any data.

## Supplementary Materials

The following are available online at https://www.mdpi.com/article/10.3390/ijerph182111500/s1, Table S1: Search strategies for English and Chinese databases; Table S2: Data extraction form; Table S3: List of excluded full-text studies; Table S4: PRISMA 2020 Checklist.
