# Supplementary material for: Effectiveness of Music-Based Intervention in Improving Uncomfortable Symptoms in ICU Patients: An Umbrella Review
Source: Int J Environ Res Public Health. 2021 Nov 1;18(21):11500. doi: 10.3390/ijerph182111500 (PMC8582781; doi:10.3390/ijerph182111500)
Supplement: Supplementary file 1 [file ijerph-18-11500-s001.zip › ijerph-1399428-supplementary.pdf]

Supplementary Materials

Table S1 Text: Search strategies for English and Chinese databases.

Database	Search Strategies
PubMed (78)	Search: (((“critical”[All Fields] OR “critically”[All Fields]) AND “ill”[All Fields]) OR ((“critical”[All Fields] OR “critically”[All Fields]) AND “ill”[All Fields]) OR “Critical Illness”[MeSH Terms] OR “Critical Care”[MeSH Terms] OR “Critical Care Nursing”[MeSH Terms] OR ((“critical”[All Fields] OR “critically”[All Fields]) AND “care*”[All Fields]) OR ((“intensive”[All Fields] OR “intensives”[All Fields]) AND “care*”[All Fields]) OR “Intensive Care Units”[MeSH Terms] OR “icu”[All Fields] OR ((“acute”[All Fields] OR “acutely”[All Fields] OR “acutes”[All Fields]) AND “ill”[All Fields])) AND (“music*”[All Fields] OR “Music Therapy”[MeSH Terms] OR “Music”[MeSH Terms] OR “song*”[All Fields] OR “rhythm*”[All Fields] OR (“melodies”[All Fields] OR “melodious”[All Fields] OR “melody”[All Fields] OR “melody s”[All Fields]) OR (“melodies”[All Fields] OR “melodious”[All Fields] OR “melody”[All Fields] OR “melody s”[All Fields]) OR (“singing”[MeSH Terms] OR “singing”[All Fields] OR “sing”[All Fields]) OR (“singing”[MeSH Terms] OR “singing”[All Fields] OR “sings”[All Fields]) OR (“singing”[MeSH Terms] OR “singing”[All Fields]) OR (“sound”[MeSH Terms] OR “sound”[All Fields] OR “sounded”[All Fields] OR “soundings”[All Fields] OR “sounds”[All Fields] OR “sound s”[All Fields] OR “sounding”[All Fields])) Filters: Meta-Analysis, Review, Systematic Review, Humans, Adult: 19+ years<br>Sort by: Most Recent
Embase (267)	(‘intensive care unit’/exp OR ‘intensive care unit’ OR ‘critically ill patient’/exp OR ‘critically ill patient’ OR ‘artificial ventilation’/exp OR ‘artificial ventilation’ OR icu) AND (‘music’/exp OR music OR ‘music therapy’/exp OR ‘music therapy’ OR ‘auditory stimulation’/exp OR ‘auditory stimulation’ OR ‘singing voice handicap index’/exp OR ‘singing voice handicap index’) AND (‘systematic review’/exp OR ‘systematic review’ OR ‘meta analysis’/exp OR ‘meta analysis’ OR ‘review’/exp OR review)
The Cochrane Library (17)	ID Search Hits<br>#1 MeSH descriptor: [Intensive Care Units] explode all trees 3864<br>#2 MeSH descriptor: [Critical Illness] explode all trees 2466<br>#3 #1 OR #2 5636<br>#4 MeSH descriptor: [Music Therapy] explode all trees 894<br>#5 MeSH descriptor: [Music] explode all trees 697<br>#6 sound 6753<br>#7 sing 403<br>#8 #4 OR #5 OR #6 OR #7 8454<br>#9 #3 AND #8 107<br>17 Cochrane Reviews matching “#9 - #3 AND #8”
Airiti Library (1)	查詢 (音樂) = 篇名.關鍵字.摘要 AND (加護病房) = 篇名.關鍵字.摘要 AND (系統性文獻回顧) = 篇名.關鍵字.摘要

	查詢 (intensive care) = 篇名.關鍵字.摘要 AND (music) = 篇名.關鍵字.摘要 AND (systematic review) = 篇名.關鍵字.摘要查詢表達式：(([ALL3]:(intensive care) AND [ALL3]:(music)) AND [ALL3]:(systematic review))
CINAHL Complete (86)	AB ((critical ill*) OR (critically ill*) OR ("Critical Illness"[Mesh]) OR ("Critical Care"[Mesh]) OR ("Critical Care Nursing"[Mesh]) OR (critical care*) OR (intensive care*) OR ("Intensive Care Units"[Mesh]) OR (ICU*) OR (acutely ill)) AND AB ((music*) OR ("Music Therapy"[Mesh]) OR ("Music"[Mesh]) OR (song*) OR (rhythm*) OR (melody) OR (melodies) OR (sing) OR (sings) OR (singing)) AND (Meta-Analysis OR Review OR Systematic Review)<br>Set limits-age group: All Adult
ProQuest (86)	ab((critical ill*) OR (critically ill*) OR ("Critical Illness" [Mesh]) OR ("Critical Care" [Mesh]) OR ("Critical Care Nursing" [Mesh]) OR (critical care*) OR (intensive care*) OR ("Intensive Care Units" [Mesh]) OR (ICU*) OR (acutely ill)) AND ab((music*) OR ("Music Therapy" [Mesh]) OR ("Music" [Mesh]) OR (song*) OR (rhythm*) OR (melody) OR (melodies) OR (sing) OR (sings) OR (singing)) AND ab(Meta-Analysis OR Review OR Systematic Review)<br>Other restrictions-age group: middle-aged (45-64 years old), adult (19-44 years old), elderly (65 years old and above), elderly (80 years old and above)
Web of Science (80)	**Intensive Care Units OR ICU OR critically ill OR Ventilators OR acutely ill** (Abstract) and **music OR sound OR song OR sing OR sings OR singing OR melody OR melodies** (Abstract) and **Review Articles** (Document Types)
Epistemonikos (181)	(advanced_title_en:(((("critical" OR "critically") AND "ill") OR (("critical" OR "critically") AND "ill") OR "Critical Illness" OR "Critical Care" OR "Critical Care Nursing" OR (("critical" OR "critically") AND "care*") OR (("intensive" OR "intensives") AND "care*") OR "Intensive Care Units" OR "icu" OR (("acute" OR "acutely" OR "acutes") AND "ill")) AND ("music*" OR "Music Therapy" OR "Music" OR "song*" OR "rhythm*" OR ("melodies" OR "melodious" OR "melody" OR "melody s") OR ("melodies" OR "melodious" OR "melody" OR "melody s") OR ("singing" OR "singing" OR "sing") OR ("singing" OR "singing" OR "sings") OR ("singing" OR "singing") OR ("sound" OR "sound" OR "sounded" OR "soundings" OR "sounds" OR "sound s" OR "sounding"))) OR advanced_abstract_en:(((("critical" OR "critically") AND "ill") OR (("critical" OR "critically") AND "ill") OR "Critical Illness" OR "Critical Care" OR "Critical Care Nursing" OR (("critical" OR "critically") AND "care*") OR (("intensive" OR "intensives") AND "care*") OR "Intensive Care Units" OR "icu" OR (("acute" OR "acutely" OR "acutes") AND "ill")) AND ("music*" OR "Music Therapy" OR "Music" OR "song*" OR "rhythm*" OR ("melodies" OR "melodious" OR "melody" OR "melody s") OR ("melodies" OR "melodious" OR "melody" OR "melody s") OR ("singing" OR "singing" OR "sing") OR ("singing" OR "singing" OR "sings") OR ("singing" OR "singing") OR ("sound" OR "sound" OR "sounded" OR "soundings" OR "sounds" OR "sound s" OR "sounding")))) [Filters: classification=systematic-review, protocol=no]

Table S2: Data extraction form

**Name Reviewer**	
**Cross-checked**	
**Systematic reviews title**	
First author	
Publication year	
Review objective/s	
Review question/s	
Primary studies (n)	
Primary studies countries	
Other	
**Participants**	
Total number of participants(rang) of included studies	
Female (%)	
Age (mean/ median/ Minimum -Maximum)	
Data range (publication year) of included studies	
Other	
**Music-based Interventions**	
Total number of intervention groups	
Music type	
Total number of Sessions	
Duration(min)	
Tempo (bpm)beats per minute	
Timing for intervention	
Music Selection (researcher/patient/music therapist	
Delivery of intervention group (headphones, earphones..)	
Delivery of control group (Headphones, Earphones..)	
Other	
**Outcome**	
Positive outcomes (significant result reported)	
No change outcomes	
Harm/ adverse effect	
Quality of each trial appraisal	
Summary of critical appraisal	
Other	

Table S3: List of excluded full-text studies
Characteristics of excluded studies (N = 14)

Item	Paper	Reason for Exclusion
1	Burrai, F.; Forton Magavern, E.; Micheluzzi, V.; Magnaghi, C.; Apuzzo, L.; Brioni, E., Effectiveness of Music to Improve Anxiety in Hemodialysis Patients: A Systematic Review and Meta-analysis. *Holistic Nursing Practice* **2020,** 34, (6), 324-333. https://doi.org/10.1097/HNP.0000000000000411	Insufficient reporting setting, patients undergoing HD, none clearly state whether they are ICU patients.
2	Sousa, L.; Ferreira, A. R.; Fernandes, L., P.178 Managing agitation in patients with dementia in the acute setting: medicines vs music – a scoping review. *European Neuropsychopharmacology* **2019,** 29, S136. https://doi.org/10.1016/j.euroneuro.2019.09.222	Not a correct intervention; a scoping review of most papers evaluated the use of pharmacological interventions.
3	García-Perdomo, H. A.; Montealegre Cardona, L. M.; Cordoba-Wagner, M. J.; Zapata-Copete, J. A., Music to reduce pain and anxiety in cystoscopy: A systematic review and meta-analysis. *Journal of Complementary and Integrative Medicine* **2018**. https://doi.org/10.1515/jcim-2018-0095	Not the correct setting, no ICU patients.
4	Horsten, S.; Reinke, L.; Absalom, A. R.; Tulleken, J. E., Systematic review of the effects of intensive-care-unit noise on sleep of healthy subjects and the critically ill. *BJA: The British Journal of Anaesthesia* **2018,** 120, (3), 443-452. https://doi.org/10.1016/j.bja.2017.09.006	Not the correct intervention, focus on ICU noise condition.
5	Khan, S. H.; Kitsis, M.; Golovyan, D.; Wang, S.; Chlan, L. L.; Boustani, M.; Khan, B. A., Effects of music intervention on inflammatory markers in critically ill and post-operative patients: A systematic review of the literature. *Heart & Lung* **2018,** 47, (5), 489-496. https://doi.org/10.1016/j.hrtlng.2018.05.015	Not the correct setting, music interventions mixed perioperative and ICU settings.
6	Khan, S. H.; Kitsis, M.; Emmett, T.; Wang, S.; Allen, D.; Golovyan, D.; Khan, B. A., Effects of music on delirium, and	Conference only with abstract, the same study with Item 5.

	inflammatory biomarkers in mechanically ventilated and post-operative patients: A systematic review of the literature. *American Journal of Respiratory and Critical Care Medicine* **2018,** 197.	
7	AlQahtani, R. M.; Newman, A.; Moragno, J.; Fox-Robichaud, A. E., Noise control in acute care units: A systematic review and meta-analysis of randomized controlled trials. *American Journal of Respiratory and Critical Care Medicine* **2017,** 195.10.1164/ajrccm-conference.2017.A22	Conference only with abstract, not a correct intervention, focus on noise reduction strategies.
8	Cole, L. C.; LoBiondo-Wood, G., Music as an Adjuvant Therapy in Control of Pain and Symptoms in Hospitalized Adults: A Systematic Review. *Pain Management Nursing* **2014,** 15, (1), 406-425. https://doi.org/10.1016/j.pmn.2012.08.010	Not the correct setting, mixed non-ICU and ICU hospitalized patients.
9	Pelizon, L. R.; Cruz, I. C. F. d., The effectiveness of music therapy for the treatment of anxiety; depression - Sistematic Literature Review. *Journal of Specialized Nursing Care* **2011,** 4, (1).	Abstract only; not enough information to determine music-based intervention in improving uncomfortable symptoms.
10	Moon Fai, C.; Zi Yang, W.; Thayala, N. V., A systematic review on the effectiveness of music listening in reducing depressive symptoms in adults. *JBI Library of Systematic Reviews* **2010,** 8, (31). https://doi.org/10.11124/jbisrir-2010-163	Not the correct setting, no ICU patients.
11	De Niet, G.; Tiemens, B.; Lendemeijer, B.; Hutschemaekers, G., Music-assisted relaxation to improve sleep quality: Meta-analysis. *Journal of Advanced Nursing* **2009,** 65, (7), 1356-1364. https://doi.org/10.1111/j.1365-2648.2009.04982.x	Not the correct setting, no ICU patients.
12	Gillen, E.; Biley, F.; Allen, D., Effects of music listening on adult patients' pre-procedural state anxiety in hospital. *JBI Library of Systematic Reviews* **2008,** 6, (17), 686-732. https://doi.org/10.11124/jbisrir-2008-180	Not the correct setting, no ICU patients.
13	Nilsson, U., The anxiety- and pain-reducing effects of music interventions: a systematic review. *AORN Journal* **2008,** 87, (4), 780-	Not the correct setting, music interventions focus on perioperative settings.

	807. https://doi.org/10.1016/j.aorn.2007.09.013	
14	Evans, D., The effectiveness of music as an intervention for hospital patients: a systematic review. *Journal of Advanced Nursing* **2002,** 37, (1), 8-18.	Not the correct setting, not all ICU patients; mixed pre-operative patients, outpatients undergoing procedures such as bronchoscopy.

Table S4. PRISMA 2020 Checklist

Section and Topic	Item #	Checklist item	Location where item is reported
**TITLE**			
Title	1	Identify the report as a systematic review.	P1
**ABSTRACT**			
Abstract	2	See the PRISMA 2020 for Abstracts checklist.	P1
**INTRODUCTION**			
Rationale	3	Describe the rationale for the review in the context of existing knowledge.	P2
Objectives	4	Provide an explicit statement of the objective(s) or question(s) the review addresses.	P3
**METHODS**			
Eligibility criteria	5	Specify the inclusion and exclusion criteria for the review and how studies were grouped for the syntheses.	P3
Information sources	6	Specify all databases, registers, websites, organisations, reference lists and other sources searched or consulted to identify studies. Specify the date when each source was last searched or consulted.	P3
Search strategy	7	Present the full search strategies for all databases, registers and websites, including any filters and limits used.	P3<br>Table S1
Selection process	8	Specify the methods used to decide whether a study met the inclusion criteria of the review, including how many reviewers screened each record and each report retrieved, whether they worked independently, and if applicable, details of automation tools used in the process.	P3<br>Table S3
Data collection process	9	Specify the methods used to collect data from reports, including how many reviewers collected data from each report, whether they worked independently, any processes for obtaining or confirming data from study investigators, and if applicable, details of automation tools used in the process.	P3–4<br>Table S2
Data items	10a	List and define all outcomes for which data were sought. Specify whether all results that were compatible with each outcome domain in each study were sought (e.g. for all measures, time points, analyses), and if not, the methods used to decide which results to collect.	P3<br>Table S2
	10b	List and define all other variables for which data were sought (e.g. participant and intervention characteristics, funding sources). Describe any assumptions made about any missing or unclear information.	P3<br>Table S2
Study risk of bias assessment	11	Specify the methods used to assess risk of bias in the included studies, including details of the tool(s) used, how many reviewers assessed each study and whether they worked independently, and if applicable, details of automation tools used in the process.	P4
Effect measures	12	Specify for each outcome the effect measure(s) (e.g. risk ratio, mean difference) used in the synthesis or presentation of results.	P3<br>Table S2
Synthesis methods	13a	Describe the processes used to decide which studies were eligible for each synthesis (e.g. tabulating the study intervention characteristics and comparing against the planned groups for each synthesis (item #5)).	Table S2
	13b	Describe any methods required to prepare the data for presentation or synthesis, such as handling of missing summary statistics, or data conversions.	None
	13c	Describe any methods used to tabulate or visually display results of individual studies and syntheses.	None
	13d	Describe any methods used to synthesize results and provide a rationale for the choice(s). If meta-analysis was performed, describe the model(s), method(s) to identify the presence and extent of statistical heterogeneity, and software package(s) used.	None
	13e	Describe any methods used to explore possible causes of heterogeneity among study results (e.g. subgroup analysis, meta-regression).	None
	13f	Describe any sensitivity analyses conducted to assess robustness of the synthesized results.	None

Reporting bias assessment	14	Describe any methods used to assess risk of bias due to missing results in a synthesis (arising from reporting biases).	P4
Certainty assessment	15	Describe any methods used to assess certainty (or confidence) in the body of evidence for an outcome.	Table 4
**RESULTS**			
Study selection	16a	Describe the results of the search and selection process, from the number of records identified in the search to the number of studies included in the review, ideally using a flow diagram.	Figure 1
	16b	Cite studies that might appear to meet the inclusion criteria, but which were excluded, and explain why they were excluded.	Table S3
Study characteristics	17	Cite each included study and present its characteristics.	Table 1
Risk of bias in studies	18	Present assessments of risk of bias for each included study.	Table 4
Results of individual studies	19	For all outcomes, present, for each study: (a) summary statistics for each group (where appropriate) and (b) an effect estimate and its precision (e.g. confidence/credible interval), ideally using structured tables or plots.	Table 2
Results of syntheses	20a	For each synthesis, briefly summarise the characteristics and risk of bias among contributing studies.	Table 4
	20b	Present results of all statistical syntheses conducted. If meta-analysis was done, present for each the summary estimate and its precision (e.g. confidence/credible interval) and measures of statistical heterogeneity. If comparing groups, describe the direction of the effect.	None
	20c	Present results of all investigations of possible causes of heterogeneity among study results.	None
	20d	Present results of all sensitivity analyses conducted to assess the robustness of the synthesized results.	None
Reporting biases	21	Present assessments of risk of bias due to missing results (arising from reporting biases) for each synthesis assessed.	Table 4
Certainty of evidence	22	Present assessments of certainty (or confidence) in the body of evidence for each outcome assessed.	Table 4
**DISCUSSION**			
Discussion	23a	Provide a general interpretation of the results in the context of other evidence.	P14–16
	23b	Discuss any limitations of the evidence included in the review.	P16
	23c	Discuss any limitations of the review processes used.	P16
	23d	Discuss implications of the results for practice, policy, and future research.	P17
**OTHER INFORMATION**			
Registration and protocol	24a	Provide registration information for the review, including register name and registration number, or state that the review was not registered.	P 3
	24b	Indicate where the review protocol can be accessed, or state that a protocol was not prepared.	P 3
	24c	Describe and explain any amendments to information provided at registration or in the protocol.	P 3
Support	25	Describe sources of financial or non-financial support for the review, and the role of the funders or sponsors in the review.	Title page
Competing interests	26	Declare any competing interests of review authors.	Title page
Availability of data, code and other materials	27	Report which of the following are publicly available and where they can be found: template data collection forms; data extracted from included studies; data used for all analyses; analytic code; any other materials used in the review.	None
